# Anomalous Origin of Right Coronary Artery Originating from the Pulmonary Trunk (ARCAPA): an Incidental Finding in a Patient Presenting with Chest Pain

**DOI:** 10.7759/cureus.1172

**Published:** 2017-04-17

**Authors:** Pragathi Balakrishna, Michael Illovsky, Youssef M Al-Saghir, Abdul M Minhas

**Affiliations:** 1 Graduate Medical Education, Orange Park Medical Center; 2 Cardiology, Orange Park Medical Center

**Keywords:** coronary artery, pulmonary artery, arcapa

## Abstract

Anomalous origin of the right coronary artery originating from the pulmonary trunk (ARCAPA) is a rare congenital coronary anomaly with an estimated prevalence of 0.002%. Most patients are asymptomatic and the anomaly is detected incidentally during evaluation for other problems. Occasionally, ARCAPA may lead to myocardial ischemia and/or sudden cardiac arrest.

We present a case of a 55-year-old female with a history of hypertension who presented to the emergency department with intermittent chest discomfort for three days. Laboratory results showed an elevated troponin of 0.18 ng/ml and subsequently increased to 0.39 ng/ml. The initial electrocardiogram study demonstrated sinus tachycardia with no acute changes. The patient was diagnosed with non-ST-segment elevation myocardial infarction. She underwent cardiac catheterization that showed 90% stenosis of the left main/left anterior descending artery. Reflux of contrast from the right coronary artery (RCA) ostium to the pulmonary artery was seen along with left to right collaterals with retrograde filling of the RCA. There was no significant obstruction of the RCA when viewed via left to right collaterals. Right heart catheterization and pulmonary angiography were performed which confirmed the origin of the RCA from the pulmonary trunk. The patient was referred for surgery and ligation of the aberrant RCA originating from the pulmonary artery was performed along with coronary artery bypass grafting x 2, left internal mammary artery to left anterior descending artery (LAD) and saphenous vein graft to the proximal posterior descending artery. The patient was discharged home with marked improvement of her symptoms.

Origin of the RCA from the pulmonary artery (ARCAPA) is a rare congenital malformation with a potentially malignant outcome for the patient. The majority of patients with ARCAPA remain asymptomatic. In this case report, the chest discomfort was due to occlusion of the LAD and was probably unrelated to the coronary malformation. However, sudden cardiac death has been linked to ARCAPA and therefore a corrective operation is recommended even for asymptomatic patients.

Of the surgical techniques available, which include: simple ligation of the RCA, ligation of the RCA with saphenous vein bypass grafting and re-implantation of the RCA into the aorta, the last method is believed to be superior for the restoration of myocardial blood supply. However, its long-term benefits have not been conclusively demonstrated. Therefore, in our patient, ligation of RCA with saphenous vein bypass grafting was done as it is recognized as a less traumatic surgical alternative to RCA implantation into the aorta.

## Introduction

Anomalous origin of the right coronary artery originating from the pulmonary trunk (ARCAPA) is a rare congenital coronary anomaly with an estimated prevalence of 0.002% [1]. The first case series was described by Brooks in 1885 [2], since then only 100 cases have been reported. Most patients are asymptomatic and usually, the anomalies are detected incidentally. Based on a literature review done by Modi, et al in 2010, twelve cases were diagnosed in infants ≤ one year of age, 44 cases were diagnosed in children ≤18 years of age, 17 cases were in adults >60 years of age, and in eight cases, the age was not recorded [3]. Patients with associated cardiac anomalies are diagnosed early in life compared to patients with isolated ARCAPA. Those without associated cardiac defects may present with a heart murmur, congestive symptoms, and sudden cardiac death or may remain asymptomatic. Detection of ARCAPA is usually incidental upon evaluation for other problems, for example, coronary angiography for chest pain [3].

Herein, we describe the case of a middle-aged female who presented with chest pain and was diagnosed with ARCAPA. Informed consent statement was obtained for this study.

## Case presentation

A 55-year-old African American female with a history of hypertension, presented with intermittent chest discomfort for three days. She described the pain as an “elephant” sitting on her chest. It was gradually progressing and was associated with diaphoresis. Review of systems was pertinent for reduced exertional capacity due to chest discomfort. Family history was negative for sudden cardiac death and premature coronary heart disease. She worked in a nursing home and did not smoke, drink alcohol or use any illicit drugs.

On examination, she appeared uncomfortable due to pain. Blood pressure was 134/63 mmHg and the heart rate 86 beats per minute. There was no jugular vein distention or carotid bruit in the neck. Cardiac examination was unremarkable except for tachycardia and breath sounds were equal bilaterally. Extremities were free of edema.

Home medications included atorvastatin 40 mg daily and metoprolol tartrate 25 mg twice daily.

Laboratory results showed elevated cardiac enzymes (troponin was elevated at 0.18 ng/ml and subsequently increased to 0.39 ng/ml). A complete blood count and basal metabolic panel were within normal range.

Her initial electrocardiography (EKG) demonstrated sinus tachycardia with no acute changes.

Echocardiogram revealed the normal function of right and left ventricles, ejection fraction was estimated in the range of 55% - 60%. There were no regional wall motion abnormalities. Wall thickness was mildly increased. The diagnosis was consistent with a non-ST segment elevation myocardial infarction.

**Figure 1:**
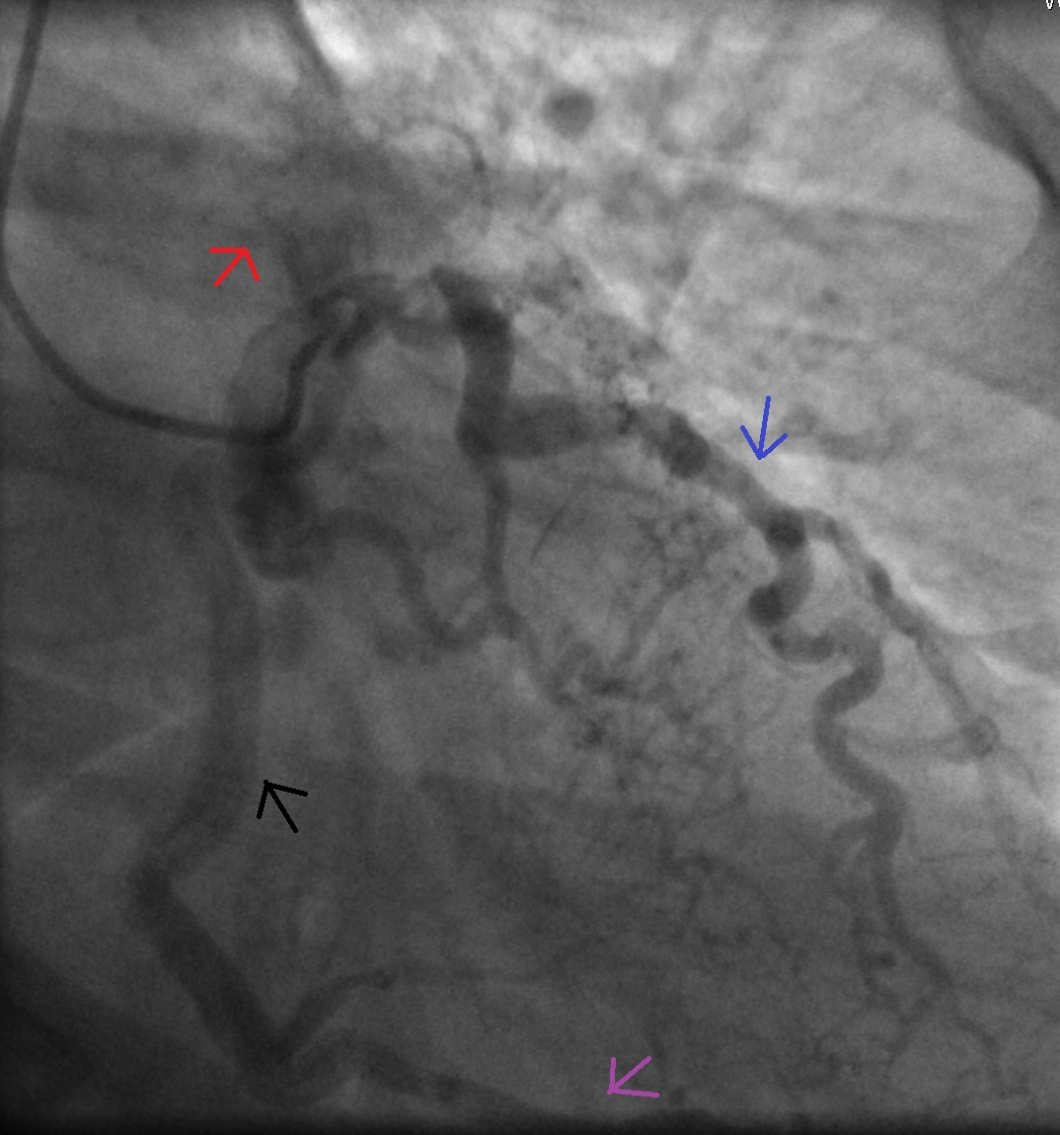
Angiogram of the left anterior descending artery (blue arrow) showing retrograde filling of right coronary artery (RCA) (black arrow), via collateral vessels (pink arrow). Note: Contrast reflux from RCA ostium to the pulmonary artery (red arrow)

The left heart catheterization showed 90% stenosis of the left main/left anterior descending artery. The right coronary artery (RCA) ostium was unidentifiable and there appeared to be left to right collaterals with retrograde filling of the RCA. The opening of the RCA appeared to be in the superior cardiac structures most likely, the pulmonary artery (Figure 1). There was no significant obstruction of the RCA when viewed via left to right collaterals. Computed tomography angiography (CTA) was done for better assessment of anatomy but the RCA origin was not well delineated. Right heart catheterization and pulmonary angiography were performed to confirm the origin of RCA. On pulmonary angiography the origin of RCA from the pulmonary trunk was evident. The visualized portion of pulmonary artery appeared patent.

Ligation of the aberrant RCA originating from the pulmonary artery was performed along with coronary artery bypass grafting x2, left internal mammary artery (LIMA) to left anterior descending artery (LAD) and saphenous vein graft to a proximal posterior descending artery (PDA).

The patient was discharged home with marked improvement of her symptoms.

## Discussion

Congenital coronary artery anomalies are rare in the general population with an incidence of only 0.3%–0.9% increasing up to 36% in patients with congenital heart disease [4]. There have been various classifications proposed based on anatomy, angiographic, and hemodynamic findings [4]. According to Greenberg, et al., major anomalies leading to abnormal myocardial perfusion are anomalous origin from the pulmonary artery, an origin of coronary artery from opposite or non-coronary sinus, myocardial bridging, and coronary artery fistula [5].

Amongst anomalous origin from the pulmonary artery, four variations of this condition have been described. First, an origin of the left coronary artery from the pulmonary artery (ALCAPA). Second, an origin of the right coronary artery from the pulmonary artery (ARCAPA). Third, an origin of an accessory coronary artery from the pulmonary artery. Fourth, an origin of the entire coronary circulation from the pulmonary artery [4]. ALCAPA is more common than ARCAPA and is fatal in infancy [6]. The incidence of ARCAPA is estimated to be 0.002% of the population and represents 0.12% of coronary anomalies [1]. The age of the patients when the abnormality was recorded ranged from day one to 90 years of age. As many patients remain asymptomatic, the true incidence of this coronary malformation might be higher. In contrast, the Bland–White–Garland syndrome (BWGS), patients often show mitral insufficiency and signs of anterolateral myocardial infarction early in childhood [7]. When symptomatic, the clinical presentation of patients with an ARCAPA is non-uniform, including dyspnea (17%), fatigue (13%), congestive heart failure (30%), angina (17%), myocardial infarction (nine percent), and even sudden cardiac arrest (17%) [7]. The time of onset and severity of symptoms depends on the type of anomaly, the direction of blood flow in the anomalous vessel and extent of collateralization. Normally, there is a retrograde flow in the anomalous artery due to the pressure difference between systemic and pulmonary circulation leading to inter coronary steal phenomena. Any increase in oxygen demand leads to exhaustion of the physiologic reserve resulting in ischemia or infarction leading to sudden cardiac arrest [6].

EKG in ARCAPA may be normal or it may show left ventricular hypertrophy or deep Q-waves in the inferior leads. Of 57 previously reported cases, the abnormal vessel was first diagnosed during angiography in 30 patients [7]. Other diagnostic modalities include cardiac computed tomography, coronary angiogram and cardiovascular magnetic resonance which provide excellent visualization of coronary artery anomalies and provide detailed anatomic information of the origin, course, and relationship of the anomalous coronary artery [7].

As adverse outcomes including increased risk of myocardial infarction and sudden cardiac death have been described in ARCAPA patients regardless of symptoms, surgical correction is recommended whenever this anomaly is diagnosed [8]. Surgical techniques available include simple ligation of the RCA, ligation of the RCA with saphenous vein bypass grafting, and re-implantation of the RCA into the aorta [9]. The aim of surgical correction is to eliminate the left-to-right shunt and establish dual coronary circulation to prevent the potential risk of myocardial ischemia from coronary steal. The location of the ostium of the right coronary artery in the pulmonary artery will influence the technique used for surgical repair [4]. Transfer of the anomalous vessel to the aorta is the treatment of choice since it provides establishment of bi coronary circulation that allows for normalization of coronary flow reserve and for greater protection against secondary coronary bypass graft changes because of age and atherosclerosis [10]. When anatomical considerations preclude re-implantation of the RCA into the aorta, ligation of the abnormal pulmonary origin of the coronary artery or arterial bypass grafting should be considered as alternative therapeutic options [5]. However, surgical and pathological reports describe the anomalous RCA as being thin- walled, dilated and vein like structured; therefore, it is thought that the RCA does not tend to normalize in diameter after surgical correction of ARCAPA [9]. Also, symptoms and myocardial ischemia persisted in patients after reimplantation along with dilated coronary arteries with persistent slow runoff into the periphery [9]. Hence, in our patient ligation of RCA with saphenous vein bypass grafting was done as it is recognized as a less traumatic surgical alternative.

## Conclusions

The case described herein describes the incidental findings of a rare coronary anomaly in a patient who presented with chest discomfort. Her chest discomfort was due to occlusion of the left anterior descending artery (LAD) and was probably unrelated to the coronary malformation. Sudden cardiac death has been linked to the anomalous origin of the right coronary artery originating from the pulmonary trunk (ARCAPA) and therefore a corrective operation is recommended regardless of symptom status.

## References

[1] Williams IA, Gersony WM, Hellenbrand WE (2006). Anomalous right coronary artery arising from the pulmonary artery: a report of 7 cases and a review of the literature. Am Heart J.

[2] Brooks HSJ (1885). Two cases of an abnormal coronary artery of the heart arising from the pulmonary artery: with some remarks upon the effects of this anomaly in producing cirsoid dilatation of the vessels. H.S.J. Trans. of. Acad. of Med.

[3] Gupta R, Marwah A, Shrivastva S (2012). Anomalous origin of right coronary artery from pulmonary artery. Ann Pediatr Cardiol.

[4] Hakim K, Boussaada R, Hamdi I (2014). Anomalous origin of the right coronary artery from the pulmonary artery. Two case reports. The Egyptian heart journal.

[5] Greenberg MA, Fish BG, Spindola-Franco H (1989). Congenital anomalies of the coronary arteries. Classification and significance. Radiol Clin North Am.

[6] Parasramka S, Dufrense A (2012). Anomalous origin of right coronary artery from pulmonary artery presenting as chest pain in a young man. J Cardiol Cases.

[7] Radke PW, Messmer BJ, Haager PK (1998). Anomalous origin of the right coronary artery: preoperative and postoperative hemodynamics. Ann Thorac Surg.

[8] Kuhn A, Kasnar-Samprec J, Schreiber C (2010). Anomalous origin of the right coronary artery from the pulmonary artery (ARCAPA). Int J Cardiol.

[9] Kautzner J, Veselka J, Rohac J (1996). Anomalous origin of the right coronary artery from the pulmonary trunk: Is surgical reimplantation into the aorta a method of choice. Clin Cardiol.

[10] Luciani GB, Vendrametto F, Barozzi L (2006). Repair of anomalous right and circumflex coronary arteries arising from the pulmonary artery. J Thorac Cardiovasc Surg.

